# On Dislocations of the Shoulder, Reduced by Manipulation

**Published:** 1867-02

**Authors:** D. G. Rush

**Affiliations:** Chicago


### CHICAGO MEDICAL JOURNAL.

Vol. XXIV. FEBRUARY, 1867. No. 2.

### ORIGINAL CONTRIBUTIONS.

### *On Dislocations of the Shoulder, reduced by Manipulation.* By D. G. Rush, M.D., of Chicago.

Dislocations of the shoulder-joint being admitedly more frequent than luxations of all the other joints of the human frame, and, with one exception, most difficult to reduce, it is desirable that some means of reduction, safe and reliable, should be devised and taught. Such means, it is confidently believed, exists in the shoulder articulation itself; the surgeon needs only call them into requisition; the humerus can be made to serve as a powerful lever, and the ligaments to maintain a steady fulcrum at an eccentric point with the head of the humerus and the brim of the glenoid cavity.

I will briefly instance the modes now generally adopted before claiming the necessity of their being superseded by others. Systematic writers on surgery almost universally insist on the application of direct traction force, in reductions generally, either by manual or instrumental means. The first expedient being to pull forcibly at the arm in a line with the body, the surgeon's heel being pressed against the scapula in the axilla. This failing, as it not unfrequently will, more especially in cases where the neck of the humerus is tightly embraced by the rent edges in the capsular ligament; powerful force is applied to the limb whilst it is carried in various directions; first at a right angle with the body, then vertically, with the arm brought to the side of the patient's head, as originally taught, many years ago, by Dr. White.

All these efforts failing, for they have, and may continue to fail, at the hands of skillful surgeons, the same line of procedure is prosecuted with increased heroism, and the pulleys are now substituted for manual force. With this powerful instrument failure does not seem possible, as every resisting element is at the mercy of the surgeon. We cannot fail to notice, however, the reluctance with which they are resorted to, or advised by surgeons most experienced in their use. Great injuries, indeed, have resulted from their use, such as rupture of the axillary artery in chronic, and fracture of the border of the glenoid cavity in recent cases; and, as in a case brought to my notice, the skin parting where the pulleys were secured to the arm by a wet roller, a ghastly wound resulted, and a suit for malpractice was instituted. From the evidence, too, that recoveries following the use of the pulleys are frequently slow and not satisfactory, it is justly inferable that many injuries result from their employment which do not at first manifest themselves, or for prudential reasons are not published.

That there is a want, then, to be supplied in this department of surgery, I will now accept as generally conceded. In demonstration of the practical value of the reduction of the shoulder-joint by manipulation, an operation which, it is believed, supplies the above want, the following case is presented in illustration. Similar cases came under my notice in the military service, which had resisted protracted efforts, and were readily reduced by the simple movements shortly to be described.

On the night of December 21st, 1866, I was called to see Mr. F., of Chicago. I was informed by the medical attendant that Mr. F. had met with an accident which resulted in the dislocation of the shoulder, and that because of the rigidity of the muscles, it could not be reduced. On examination I found a dislocation downwards, the head of the humerus occupying the axilla. Protracted and various efforts had been made to effect a reduction; several persons pulling while the surgeon's foot occupied the axilla. After these, my services were solicited.

Finding the parts very painful, and the patient unwilling to endure the pain attending the slightest movements of the arm, chloroform was administered before any efforts at reduction were repeated. When under the full effects of chloroform, the forearm bent upon the arm; the humerus was firmly rotated outwards. This rotary tension being preserved, the elbow was brought to a right angle with the body, and with a reverse rotary movement, the arm was then carried across the chest on a line with the clavicle, an audible snap indicating the return of the head of the humerus to the deserted cavity.

It is not deemed necessary to consider, in this article, any of the causes which may produce the injury under consideration, the several varieties, nor the symptoms by which it may be recognized, as they are generally unequivocal and readily distinguished. I may, however, remark in this connection, that the reduction of the shoulder-joint by manipulation, is equally applicable to all the varieties of shoulder dislocations, modified according to the situation of the rupture in the ligaments.

Reduction of the shoulder-joint by manipulation is not a new discovery, attention having been called to it as early as 1833, by Sir Philip Crampton, who strongly advocated the operation, and submitted rules for its performance. These first rules did not seem to succeed well in other hands, for they were based on the fatal supposition that all the surgeon need aim to accomplish was to first induce relaxation of the muscles on the dorsum of the scapula, and then by reverse movement of the arm excite contraction of the same. This operation consequently had fallen quite into disuse, when, after the discovery of the reduction of the hip-joint by manipulations by Dr. Reed, of New York, and the superiority of his procedure had been fully acknowledged, it was revived by Henry H. Smith, of Philadelphia, who repeatedly demonstrated the practical value of the plan, on the shoulder, not less than on the hip-joint.

In laying down rules, however, for the performance of these manipulatory movements, Dr. Smith, too, fell into the error of supposing that the reduction depended on the responses induced in the posterior scapular muscles. Although Dr. Smith directs supination and pronation of the hand to be made during the several steps of the operation, yet his conclusions as to the cause of reduction so impress every one with the idea that the above-named muscles, in some way, must be alone instrumental in restoring the injury, that he naturally neglects to attend to the most important principles; and failing in his first efforts, the operation is suspended. Hence, the vain effort this operation has hitherto made to receive its merited rank and appreciation. It is confidently believed, that the philosophy of these manipulations need only be clearly comprehended to place this operation in the first rank as a surgical procedure.

It is not strange that the theory of muscular influence should adhere so tenaciously to the subject of dislocations, for from the earliest teachings on surgery we see nothing but a war of surgeon and muscle arrayed against each other. Much stress has been laid on the action of the deltoid and spinati muscles pressing the head of the humerus firmly against the lower border of the neck of the scapula, while the pectoral and others draw the humerus inwards.

Now, whilst it is not alleged that the muscles are not capable of offering any opposition to our efforts, I am of opinion that their resistance is, comparatively, quite subordinate; so are they also equally ineffectual as an aid in these manipulations. Chloroform is a certain test of these theories, for it is acknowledged on all sides that it relaxes the muscles completely. If the muscular theory were true, this potent agent should remove every obstacle, and the displaced member be freely movable in a line with the displacement. Though it generally facilitates the reduction by traction, yet it often fails to remove the difficulty. Again, if it were true that the reduction by manipulation was effected by causing certain muscles to respond to our will, chloroform must necessarily defeat our object. That it does not defeat it, I have myself too frequently demonstrated to admit of doubt. I shall now attempt to make the operation intelligible.

We will suppose we are called upon to reduce a dislocation of the shoulder downwards. The patient lying on a table, the affected side brought to the edge, and chloroform administered, the surgeon grasps the wrist with one hand and the elbow with the other. Bringing the forearm to a right angle with the arm, the elbow is pressed against the side of the body, and the hand drawn outwards, thus producing rotation of the humerus, and tension of the capsular ligament; the tension being maintained, the elbow is brought to a right angle with the body and slightly depressed. The arm is next carried across the chest in a line with the clavicle, reversing the rotation of the humerus in this last movement, when an audible snap will generally indicate the reduction of the dislocation. Should the head of the bone, by any chance, slide back into the axilla, these movements must be repeated.

In order to make these manipulations more comprehensive, a few remarks may serve to elucidate them. The forearm is bent on the arm, simply to afford a lever to facilitate the rotation of the humerus, which is rotated for the double purpose of producing tension of the ligaments whilst the arm is abducted, and bringing the tubercle down to rest against the border of the glenoid cavity, where it becomes arrested and acts as a fulcrum. The head of the humerus being eccentric to the fulcrum, it is not difficult to understand how it may be made to slip over the brim of the cavity, and returned to its normal lodgement.

